# Radiation–Sensitive Thin Film Dosimeter Based on Polyvinyl Alcohol (PVA)/Hafnium Dioxide (HfO_2_)/Silver Nitrate (AgNO_3_) Composite: Colorimetric Characterization and Dose–Response Analysis for Low–Dose Gamma–Ray Applications

**DOI:** 10.3390/polym18172165

**Published:** 2026-09-04

**Authors:** Saleh Alashrah

**Affiliations:** Department of Physics, College of Science, Qassim University, Buraydah 51452, Qassim, Saudi Arabia; ashrh@qu.edu.sa

**Keywords:** gamma–ray, dosimetry, colorimetric, X–ray diffraction, PVA/HfO_2_/AgNO_3_, Kubelka–Munk, CIELAB, CMYK analysis

## Abstract

The development of sensitive, low–cost, and visually readable dosimeters for low gamma–ray exposures is important for occupational and environmental radiation monitoring and for other low–dose applications. This work investigates a colorimetric and optical thin–film dosimeter based on polyvinyl alcohol (PVA) containing silver nitrate (AgNO_3_) and hafnium oxide (HfO_2_). The film was fabricated using a solution–casting technique. The dosimetric response was evaluated over an absorbed–dose range of 22.2–65.2 mGy using diffuse reflectance spectroscopy, Kubelka–Munk (K/S) analysis, CIELAB colorimetry, CMYK image–based analysis, and X–ray diffraction (XRD). Irradiation produced a dose–dependent decrease in visible reflectance and a corresponding increase in optical absorption. The K/S response increased with dose, while CIELAB analysis showed a systematic decrease in lightness and an increase in total color difference (${\Delta E}_{ab}^{*}$), reaching approximately 25 at 65.2 mGy. Linear regression of ${\Delta E}_{ab}^{*}$ over 0–65.2 mGy gave y = 0.399x − 1.0029 with R^2^ = 0.9845. CMYK analysis also showed a clear dose response, with the yellow channel (ΔY) exhibiting the largest relative change among the chromatic channels. XRD identified monoclinic HfO_2_ as the dominant crystalline filler phase and showed dose–associated changes in peak intensity, peak position, and the relative prominence of the broad PVA–related feature. At the highest XRD dose, several HfO_2_ reflections weakened while the broad contribution near 2θ ≈ 19.9–20° became more prominent. These changes are interpreted as dose–dependent structural modification and partial loss of resolved crystalline order rather than definitive evidence of a newly formed crystalline phase. A surface morphology and microstructure analysis was performed on control and γ–ray–irradiated PVA/HfO_2_/AgNO_3_ nanocomposite films using scanning electron microscopy (SEM–EDX). The morphological transition to fibrous, tree trunk–like structures seen by SEM is well correlated with the dose–dependent change in composition to higher surface Ag content, supporting the idea that radiation–induced Ag nanoparticle nucleation and growth is the primary degradation mechanism in the irradiated films. The combined optical and colorimetric results demonstrate a measurable response of the PVA/HfO_2_/AgNO_3_ formulation in the investigated low–mGy gamma–ray range.

## 1. Introduction

In many different fields, such as environmental surveillance, industrial processing, radiotherapy, and diagnostic medical imaging, precise assessment of absorbed radiation dosage is essential [1]. The accurate measurement of radiation exposure has a direct bearing on public health and safety rather than just a technical problem [1]. Accurate dosimetry is considered a key component of the radiation protection framework by radiological protection organizations like the International Atomic Energy Agency (IAEA) [2], the International Commission on Radiological Protection (ICRP) [3], and the National Council on Radiation Protection and Measurements (NCRP) [4]. The increasing use of advanced medical imaging modalities like computed tomography (CT) and image–guided interventional treatments, which often involve multiple exposures to patients in the milligray range, requires the highest measurement precision [5]. The workers in clinical and occupational radiation monitoring are still using conventional passive dosimetry devices, including thermoluminescent dosimeters (TLDs), optically stimulated luminescence dosimeters (OSLDs), alanine detectors, and radiochromic films [6,7]. Each of these technologies, however, has intrinsic drawbacks that reduce their efficacy in low–dose situations [7]. These limitations include energy dependency, poor spatial resolution, multi–step or delayed readout operations, limited reusability, and reliance on specialist equipment [8]. Specifically, for medium to high doses, radiochromic films are often more accurate. However, due to flatbed scanners’ unstable light source, they are usually linked to low sensitivity and large measurement uncertainty in the diagnostically relevant milligray zone [9].

Colorimetric and optical dosimetry have attracted increasing attention as passive detection approaches [7,10]. Ionizing radiation can cause persistent and measurable changes in optical properties such as absorption, reflectance, and visible color [11]. These changes can be quantified using spectrophotometric measurements or image–based analysis [10,12]. An important advantage is that the material itself can retain a visible record of irradiation without requiring continuous electronic readout, allowing both quantitative and qualitative assessment [12]. Such systems may therefore be useful where simple, low–cost, and distributable monitoring is desirable [7].

The Kubelka–Munk (K/S) formalism, which connects the observed diffuse reflectance to the solid–state material’s effective absorption and scattering coefficients, provides a broad explanation for the radiation–induced optical changes [13]. This method offers a quantifiable way to monitor radiation exposure’s chemical and electrical consequences. When combined with colorimetric analysis in the CIELAB color space, which characterizes color perception in terms of brightness (L*) and chromatic coordinates (a* and b*), the dosimetric response may be thoroughly defined. The metric ${\Delta E}_{ab}^{*}$ (total color difference) is especially well–suited for low–dose measurement applications and has proven beneficial in identifying subtle color changes brought on by radiation [14,15].

Inorganic metal oxides and ceramic compounds have been investigated as components of radiation–responsive composites because their composition can modify photon interactions and the local energy–deposition environment [10]. High–Z materials such as Hf–containing compounds, tungsten–based materials, and bismuth oxides can increase photon interaction probability through photoelectric absorption and Compton scattering, depending on photon energy [6]. Zinc oxide has also been investigated for radiation–induced defect formation and charge trapping that alter optical properties [16,17,18,19]. Silver nitrate (AgNO_3_) is a radiation–sensitive component used in colorimetric systems [20]. Under irradiation, Ag^+^ can undergo radiolytic reduction and generate Ag–containing metallic species whose optical response depends on particle size, morphology, aggregation state, and the surrounding dielectric environment [21,22,23]. These radiation–induced optical changes can provide a persistent colorimetric record [24,25]. Incorporating AgNO_3_ into PVA provides a flexible solid–state host, while the addition of HfO_2_ introduces a high–Z inorganic component. In the present work, the response of the complete PVA/HfO_2_/AgNO_3_ formulation is evaluated; the individual sensitizing contribution of HfO_2_ is not isolated experimentally.

Solution casting is widely used to prepare polymer composite films because it is simple, low–cost, and compatible with thin, flexible, self–supporting materials [26]. In this approach, the polymer is dissolved to form a continuous phase, and the inorganic and radiation–sensitive components are introduced under controlled mixing before solvent evaporation. Sequential addition and prolonged stirring can promote dispersion and reduce macroscopic agglomeration, while the solute concentration and casting conditions influence film thickness and reproducibility. These features make solution casting suitable for low–cost polymer dosimeter fabrication. However, the casting procedure itself does not demonstrate microscopic elemental homogeneity, which would require direct morphological or elemental mapping.

Despite substantial progress in polymer–based radiation–responsive materials, comparatively little systematic work has focused on PVA films containing both AgNO_3_ and HfO_2_ for low–dose gamma–ray colorimetry. The objective of this study is therefore to fabricate a PVA/HfO_2_/AgNO_3_ composite thin film and characterize its structural, optical, and colorimetric response to low–mGy gamma irradiation. The performance is evaluated using diffuse reflectance spectroscopy, Kubelka–Munk analysis, CIELAB colorimetry, CMYK image–based analysis, and XRD. Particular attention is given to the measured dose–response trends and to conservative interpretation of the structural and optical changes supported by the available data.

## 2. Materials and Methods

### 2.1. Materials

The composite dosimetric films were prepared from a flexible PVA matrix containing AgNO_3_ as the radiation–sensitive colorimetric component and HfO_2_ as the high–Z inorganic additive. PVA was selected because of its film–forming ability, chemical stability, and compatibility with aqueous processing. AgNO_3_ provides a radiation–responsive component capable of undergoing radiolytic reduction with an associated change in optical appearance. HfO_2_ was included to increase the photon–interaction probability of the formulation, although the present study does not independently quantify its sensitizing contribution. Table 1 summarizes the composition and functional role of the constituents. All materials were used as received. The component fractions were selected from literature precedents and practical processing considerations. Higher filler contents tested during formulation produced poorer dispersion quality and reduced optical readability; therefore, the present composition was selected as a practical balance between filler loading and film processability. Microscopic compositional uniformity is not inferred from the preparation procedure alone.

### 2.2. Preparation of Composite Thin Films

Composite thin films were fabricated using a solution–casting technique to obtain self–supporting films with controlled composition and to promote dispersion of the added components. Initially, 6.2 g of PVA powder was dissolved in 25.0 mL of distilled water under continuous magnetic stirring at 80 °C for 2 h, yielding a clear polymer solution. The solution was then cooled to room temperature under continued stirring. HfO_2_ nanoparticles (3.0 g dispersed in 10.0 mL of distilled water) and AgNO_3_ (0.8 g dissolved in 5.0 mL of distilled water) were added sequentially under continuous stirring. This procedure was used to reduce macroscopic agglomeration and promote dispersion during casting. The final mixture was poured into clean glass Petri dishes and dried at room temperature for 48 h under dark conditions to minimize premature photoinduced changes in AgNO_3_. After solvent evaporation, the self–supporting films were carefully removed. Film thickness was measured at multiple locations using a digital thickness gauge and was approximately 100 μm with only small macroscopic variation between the measured locations. The films were cut into uniform pieces and stored in light–tight, airtight envelopes before irradiation.

### 2.3. Gamma–Ray Irradiation Procedure

Gamma irradiation was performed using a ^137^Cs source (Eγ = 662 keV). A calibrated RaySafe 452 survey meter (Fluke, Everett, WA, USA) was used to determine the dose rate at the sample position, which was approximately 30 μSv·h^−1^. The administered dose levels were obtained by varying the exposure time at a fixed source–to–sample distance using the same irradiation and calibration approach as in previous work [15,24,27]. The expected uncertainty in the administered dose is approximately ±10%, based on the survey–meter calibration and irradiation reproducibility. The investigated dose range was 22.2–65.2 mGy, with unirradiated films (0 mGy) stored under the same light–tight conditions as the irradiated samples. All irradiations were performed using the same geometry and controlled experimental conditions.

### 2.4. Optical and Colorimetric Measurements

Diffuse reflectance spectra of irradiated and unirradiated films were recorded over the wavelength range of 400–700 nm using a calibrated spectrodensitometer. Radiation–induced optical changes were evaluated using the Kubelka–Munk (K/S) formalism [13].

(1) $$K/S=\frac{{\left(1-R\right)}^{2}}{2R}\;$$

where R is the measured diffuse reflectance, K is the absorption coefficient, and S is the scattering coefficient. The colorimetric measurements were conducted on a 3NH YD5010 spectrodensitometer under standard conditions (2° standard observer, D50 illuminant, and M1 measurement mode). The Commission Internationale de l’Éclairage (CIE L*a*b*) color measurements were obtained using the SDQC software, Version 1.25.1.2, that comes with the equipment [27]. The total color difference (Δ*E*∗_*ab*_) was calculated using the following equation:

(2) $${∆E}_{ab}^{*}=\sqrt{{\left(\Delta L^{*}\right)}^{2}+\;{\left({\Delta a}^{*}\right)}^{2}+\;{\left({\Delta b}^{*}\right)}^{2}}$$

where Δ terms are the differences between irradiated and unirradiated films.

CMYK colorimetric analysis was also performed using high–resolution scanned images acquired under controlled illumination conditions. Cyan (C), magenta (M), yellow (Y), and black (K) channel intensities were extracted using image–processing software (SDQC software). Dose–dependent changes (Δ*C*, Δ*M*, Δ*Y*, and Δ*K*) were evaluated relative to unirradiated films.

### 2.5. Measurement Repeatability and Data Analysis

For each dose level and irradiation, measurements were obtained from repeated readings of each condition. For the diffuse–reflectance (K/S) measurements, five replicate readings per dose were retained, and the K/S spectra are reported as the mean of the per–replicate K/S values with the corresponding standard deviation (SD). For the CIELAB (L*, a*, b*, ${∆E}_{ab}^{*}$) and CMYK metrics, only the averaged values were retained in the processed dataset, and the individual replicate readings are not available; these dose–response plots, therefore, present mean–value trends, and this is acknowledged as a limitation. For a set of *n* replicate readings *x*_i_, the mean $\bar{x}$ and SD were computed as the following equations:

(3) $$\bar{x\;}=\;\frac{1}{n}\;\sum_{i=1}^{n}x_{i}$$

(4) $$\mathrm{SD}=\sqrt{\frac{1}{n-1}\sum_{i=1}^{n}{(x}_{i}-\bar{x}{)}^{2}\;}\;$$

Dose–response linearity in the low–dose region was evaluated using least–squares regression, and the coefficient of determination (*R*^2^) is reported.

### 2.6. X–Ray Diffraction (XRD) Analysis

The crystalline structure of the composite thin films was characterized by X–ray diffraction (XRD) using a Shimadzu XRD–6000 diffractometer (Shimadzu Corporation, Tokyo, Japan) equipped with a vertical goniometer and a scintillation–counter detector. Diffraction patterns were recorded using Cu Kα radiation (λ = 1.5406 Å) at a tube voltage of 40 kV and a tube current of 30 mA. Data were collected over a 2θ range of 10–80° with a step size of 0.02° and a counting time of 0.60 s per step. The divergence slit and scattering slit were each 1°, and the receiving slit was 0.30 mm. Peak positions, d–spacings, and full width at half maximum (FWHM) values were obtained from the existing diffraction patterns where reliable peak fitting was possible. Interplanar spacings were evaluated using Bragg’s law, and crystallite–size estimates were obtained using the Scherrer relation. The diffraction data were processed using the instrument software with background correction, Kα_1_–Kα_2_ separation, and peak–search functions. Because the dominant HfO_2_ phase identified in the prepared film is monoclinic, a single cubic lattice parameter a is not an adequate descriptor; a full monoclinic unit–cell refinement was not performed, and the quantitative analysis therefore focuses on 2θ positions, d–spacings, indexed reflections, representative FWHM values, and Scherrer crystallite–size estimates.

To calculate the interplanar spacing (d–spacing), Bragg’s law was used, as shown in Equation (5):

(5) 2d sin θ = nλ

where λ = 1.54 Å is the wavelength of CuKα, θ = diffraction angle, and n = diffraction order. The relation by Debye–Scherrer was used for the mean crystallite–size calculation shown in Equation (6):

(6) $$D=\frac{\overset{`}{K\lambda }}{\beta \cos\left(\theta \right)}$$

where β is the radian FWHM, k′ ≈ 0.9 is the shape factor, and λ is the CuKα wavelength radiation. Data processing and statistical analyses were performed locally using Python 3.14.

### 2.7. SEM–EDX Analysis

The morphology of the surface of the control and γ–ray–irradiated films was assessed via SEM analysis. SEM micrographs were obtained using scanning electron microscopy (JEOL JCM–7000 LV, JEOL Ltd., Akishima, Japan) at an acceleration voltage of 15 kV. Magnifications were varied between 300× and 1000×. To improve surface conductivity, all samples were coated with a thin layer of Au prior to analysis.

## 3. Results

### 3.1. XRD Structural Analysis Results

**Figure 1:**
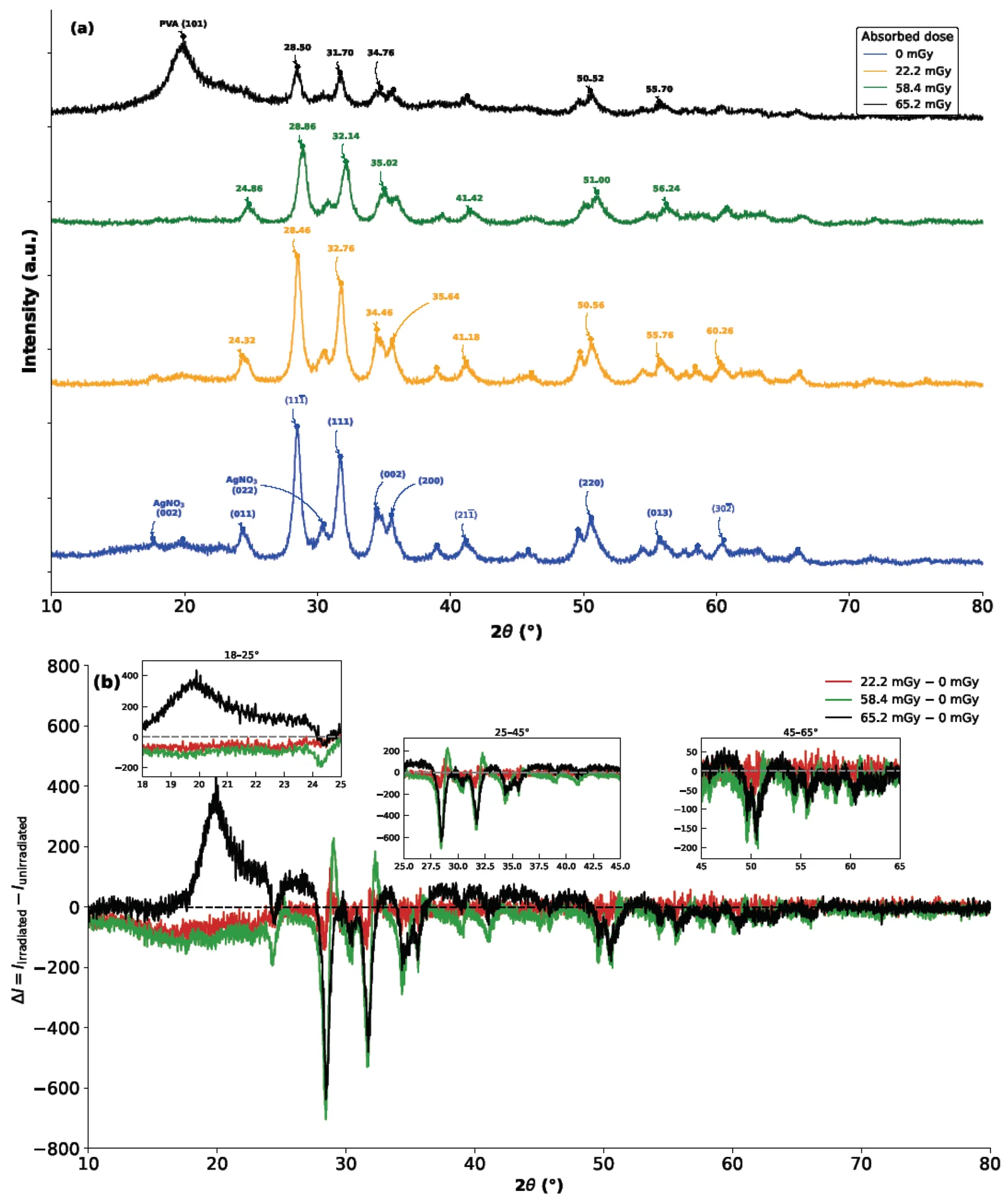
(**a**) XRD patterns of PVA/HfO_2_/AgNO_3_ composite thin films at absorbed doses of 0, 22.2, 58.4, and 65.2 mGy, plotted with vertical offsets for clarity. Major monoclinic HfO_2_ reflections, the weak low–angle feature near 17.62° in the unirradiated film, and the broad PVA–associated contribution near 2θ ≈ 19.9–20° are indicated. (**b**) Difference patterns (irradiated—unirradiated) highlighting dose–associated changes in relative diffraction intensity. The highest–dose pattern is interpreted as increased broadening and partial loss of resolved crystalline order rather than definitive formation of a new crystalline phase.

The structural response of the PVA/HfO_2_/AgNO_3_ films was examined by XRD at 0, 22.2, 58.4, and 65.2 mGy. The patterns consisted of a broad polymer–related contribution superimposed on sharper crystalline reflections. In the unirradiated film, the major reflections at 2θ ≈ 24.38°, 28.44°, 31.72°, 34.46°, 35.56°, 41.18°, 50.58°, 55.72°, and 60.54° were consistent with monoclinic HfO_2_ (space group P2_1_/c; JCPDS/PDF 34–0104; COD 1528988), with the strong reflection near 31.72° assigned to the (111) plane (Table 2). The broad feature near 2θ ≈ 19.9–20° was associated with the semicrystalline PVA matrix. The 0 and 22.2 mGy patterns showed broadly similar HfO_2_ peak positions and an estimated mean crystallite size of approximately 11.6 nm, indicating that no major HfO_2_ structural change was resolved at the lowest investigated dose. A weak low–angle reflection near 17.62° was observed in the unirradiated pattern and was tentatively assigned to an AgNO_3_–containing crystalline contribution by comparison with the cited crystallographic database (Figure 1a). This feature was not resolved after irradiation. Its disappearance was consistent with irradiation–induced modification or depletion of the corresponding AgNO_3_–containing crystalline contribution; however, XRD alone does not demonstrate complete conversion of Ag^+^ to metallic Ag^0^. At 58.4 mGy, selected HfO_2_ reflections showed modest changes in position and intensity, and the mean HfO_2_ crystallite–size estimate decreased to approximately 9.9 nm. A reflection near 51° showed pronounced broadening (FWHM ≈ 1.6°, Scherrer estimate ≈ 5.50 nm), indicating that the degree of broadening was not identical for all reflections. At 65.2 mGy, the reflection near 60.54° was no longer resolved, the reflections near 35.56° and 41.18° were weakened, and the broad PVA–associated contribution near 19.9–20° became the most prominent feature (Figure 1a,b). The apparent size estimate of 3.84 nm associated with this broad feature should not be interpreted as a mean HfO_2_ crystallite size. Overall, the higher–dose pattern is interpreted conservatively as a redistribution of diffraction intensity, increased broadening, and partial loss of resolved crystalline order rather than definitive evidence for formation of a new crystalline phase. The apparently abrupt change at 65.2 mGy should also not be taken as proof of a discrete structural threshold because only four XRD dose levels were measured. The major baseline d–spacings and phase assignments are summarized in Table 2, while the available dose–dependent structural metrics are summarized in Table 3. HfO_2_ optical absorption was reported mainly in the UV region [28,29]; thus, direct HfO_2_ absorption is unlikely to account for the dominant irradiation––associated changes observed above 500 nm. The long–wavelength response is discussed separately below in relation to the Ag–containing component and the composite environment.

### 3.2. Optical Response and Diffuse Reflectance Behavior

**Figure 2:**
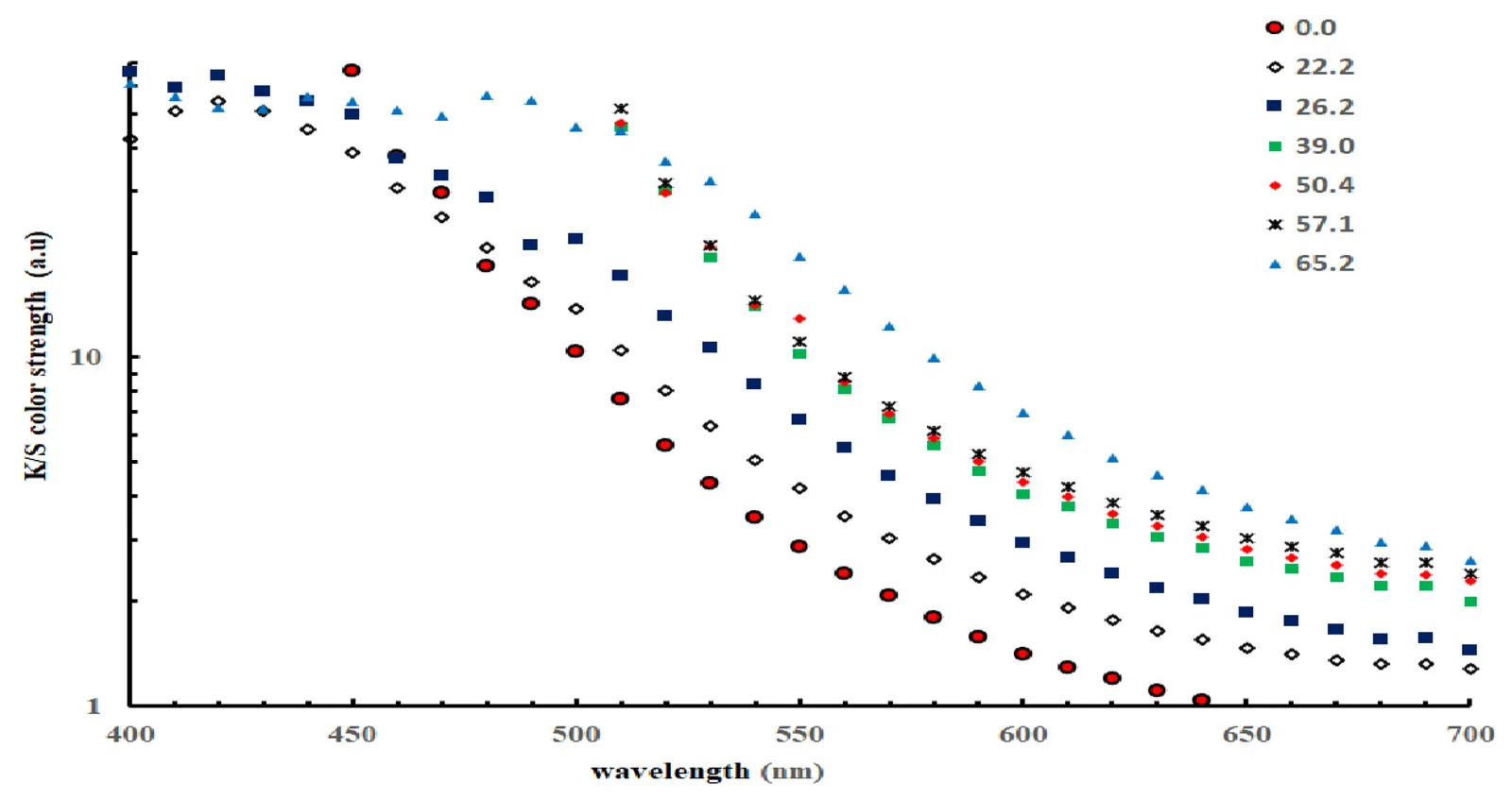
Kubelka–Munk color strength (K/S) spectra of the PVA/HfO_2_/AgNO_3_ composite film under the investigated irradiation geometry at absorbed doses of 0.0, 22.2, 26.2, 39.0, 50.4, 57.1, and 65.2 mGy, showing dose–dependent changes in optical absorption across 400–700 nm. Curves represent the mean of five replicate readings; variability is reported as ±1 SD (*n* = 5).

Diffuse reflectance spectra of the PVA/HfO_2_/AgNO_3_ composite thin films were recorded over 400–700 nm to evaluate irradiation–induced optical changes. Reproducible differences were observed between unirradiated and irradiated films, with reflectance decreasing most clearly in the 550–700 nm region (Figure 2). The localized surface plasmon resonance (LSPR) of small, isolated spherical Ag nanoparticles was often reported near 400 nm, but its spectral position was not fixed. It depends on particle size, morphology, aggregation state, and the dielectric environment. Bekhit et al. [30] reported irradiation–associated evolution of Ag nanostructures with a broad band extending to approximately 489 nm, while Djeddou et al. [31] reported components near 512.8 and 564.0 nm for polydisperse or aggregated Ag nanoparticles. Soliman et al. [21] likewise showed that the optical response of radiation–generated Ag nanoparticles depends on the resulting particle population and matrix. These reports provide a reasonable basis for describing the >500 nm absorption increase in the present composite as consistent with irradiation–induced formation or evolution of Ag–containing metallic species. However, the present optical spectra do not uniquely establish particle size, morphology, or chemical state, and the absence of an irradiated PVA/HfO_2_ control without AgNO_3_ means that a contribution from irradiation–induced changes in the PVA/HfO_2_ matrix cannot be completely excluded. HfO_2_ itself is not expected to show strong intrinsic absorption in this visible range based on the cited optical literature [28,29]. The optical response was quantified using the Kubelka–Munk (K/S) formalism. K/S changes are detectable at the lowest tested dose of 22.2 mGy and increase across the investigated dose range, reflecting increased absorption by radiation–induced absorbing species within the composite. The complete formulation therefore shows a clear dose–dependent optical response, while component–specific mechanistic assignments are treated as evidence–consistent rather than definitive.

### 3.3. CIE Lab Parameters for Colorimetric Analysis

**Figure 3:**
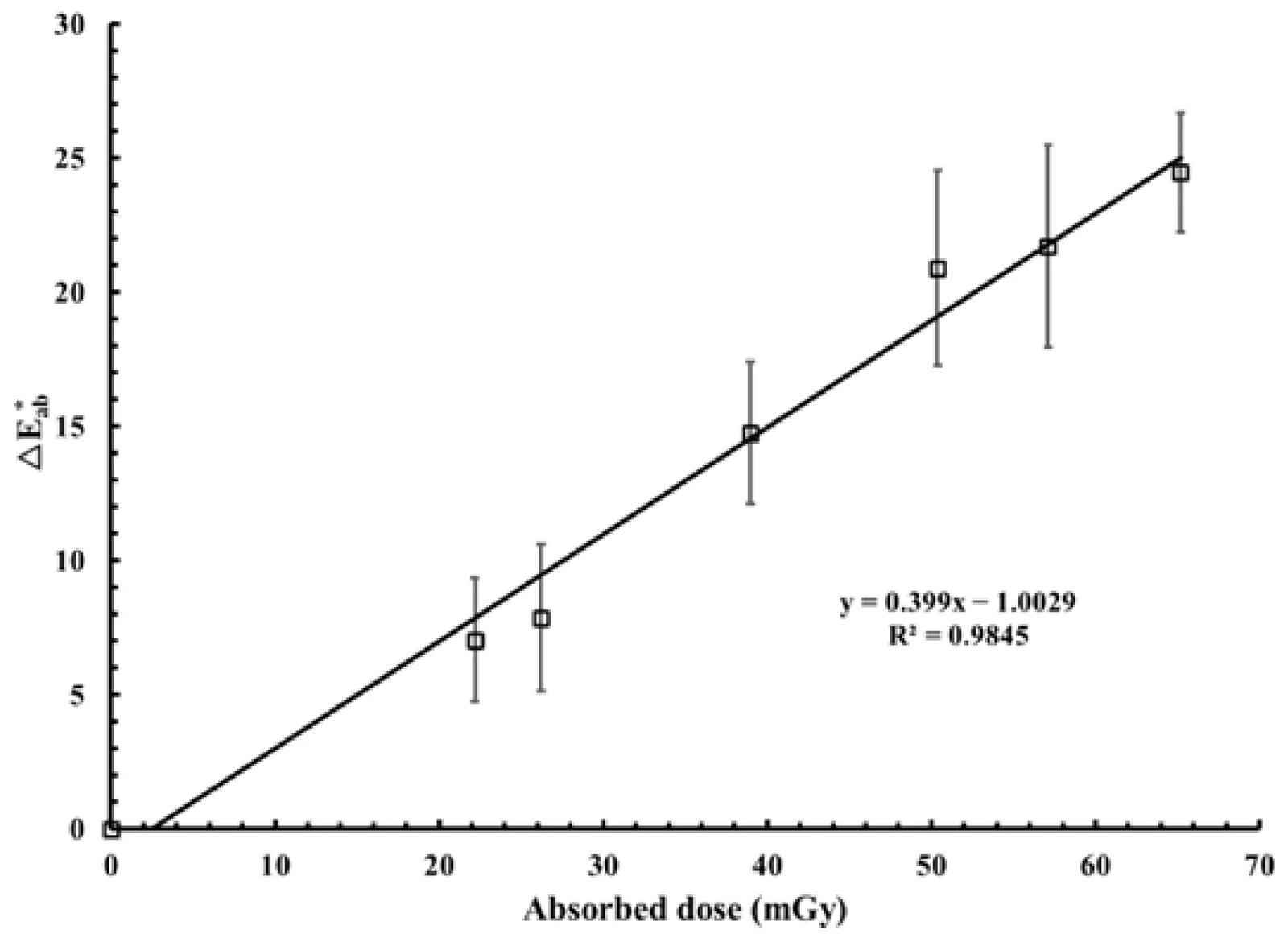
Total color difference ${\Delta E}_{ab}^{*}$ as a function of absorbed dose for the PVA/HfO_2_/AgNO_3_ composite film. Points represent the available mean values from repeated measurements. The regression line is shown over the investigated dose range.

**Figure 4:**
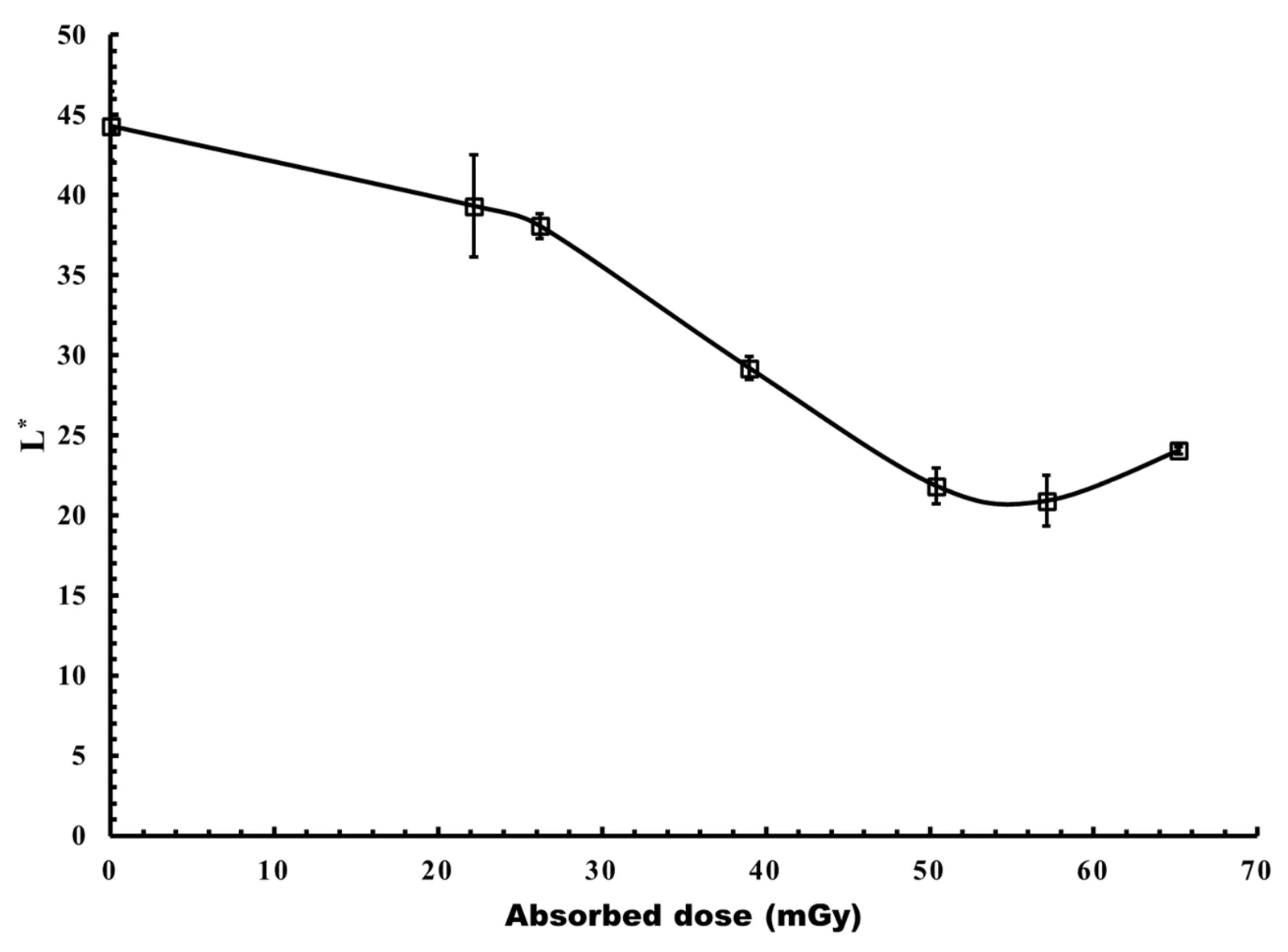
Lightness parameter (L*) of the PVA/HfO_2_/AgNO_3_ composite films versus absorbed gamma dose (0–65.2 mGy). L* decreases gradually from 44.3 at 0 mGy to a minimum of 20.9 at 57.1 mGy, then increases slightly to 24.0 at 65.2 mGy, indicating a dose–dependent darkening of the film, which is nearly monotonic in the range studied.

**Figure 5:**
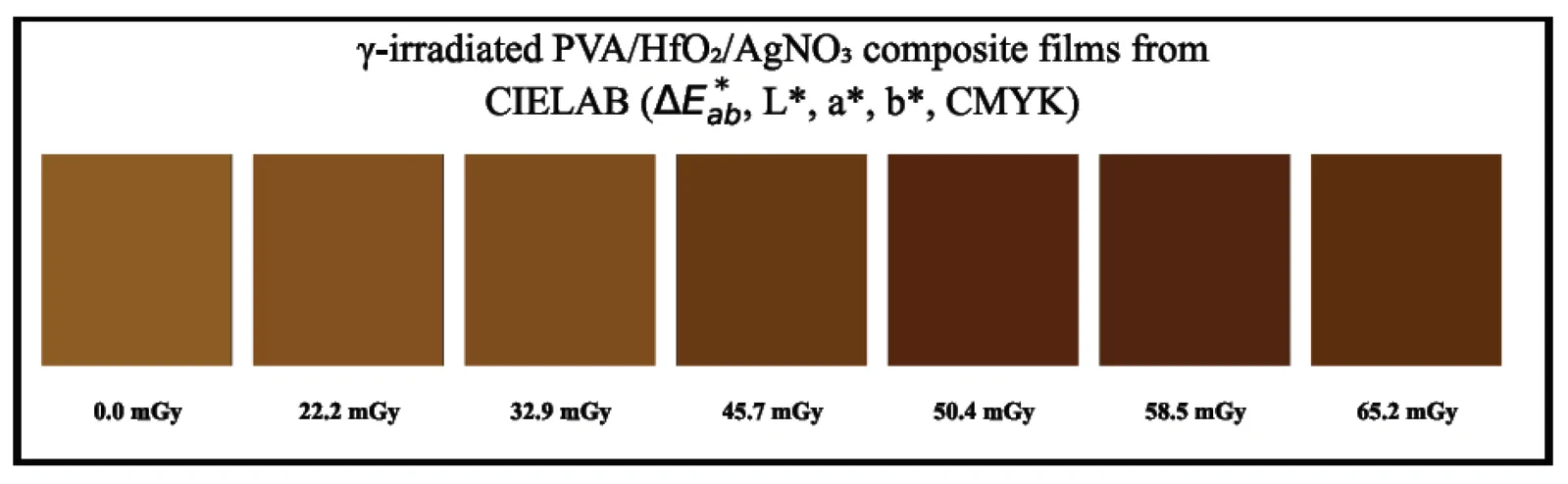
Visual color evolution of the *γ*–irradiated PVA/HfO_2_/AgNO_3_ composite film reconstructed from measured CIELAB ($∆E_{ab}^{*}$, L*, a*, b*, and the CMYK channels) for absorbed doses of 0, 22.2, 32.9, 45.7, 50.4, 58.5, and 65.2 mGy.

**Figure 6:**
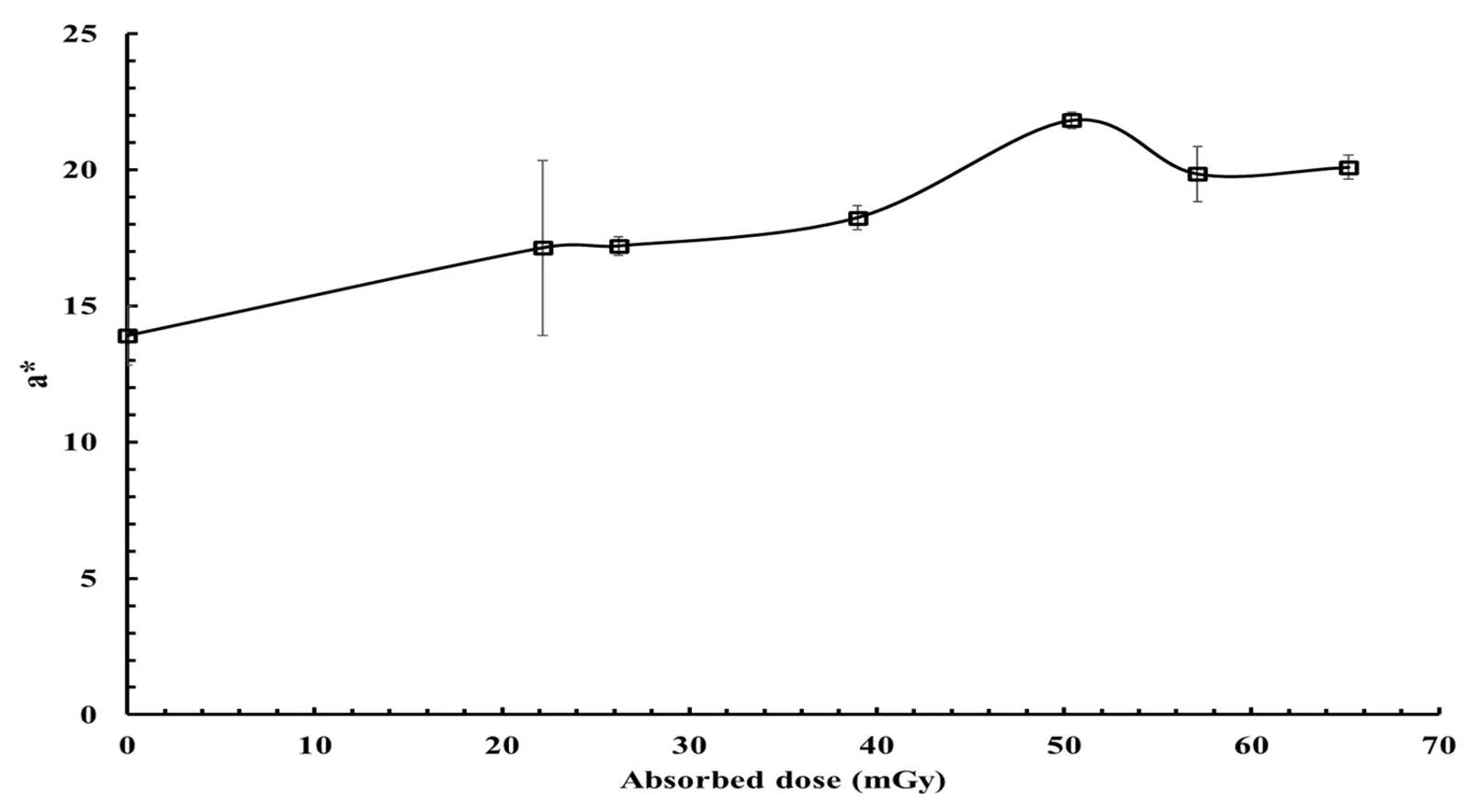
Variation in the CIELAB chromatic coordinate a* as a function of absorbed dose. Points represent mean values obtained from repeated measurements.

**Figure 7:**
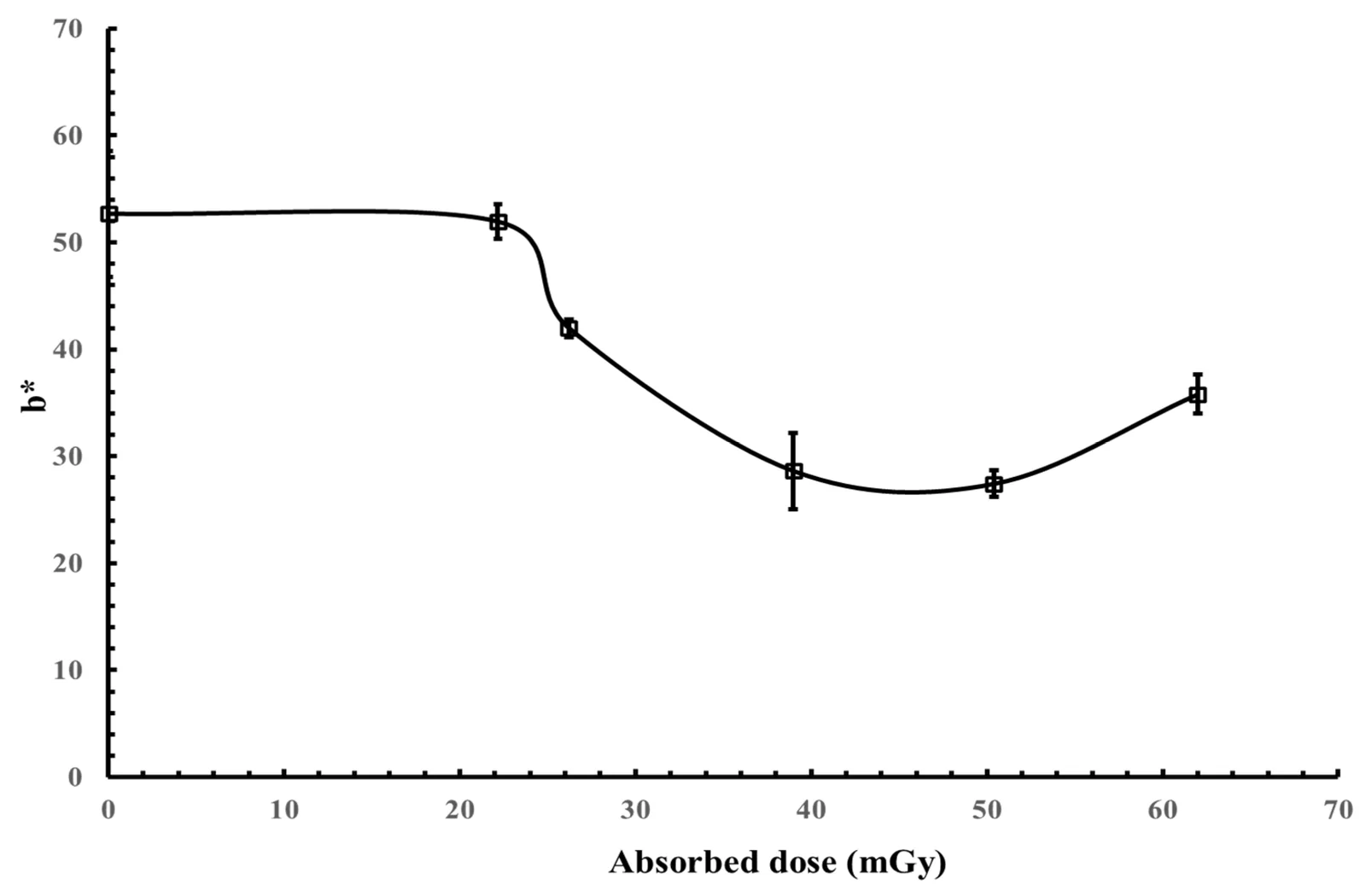
Variation in the CIELAB chromatic coordinate b* as a function of absorbed dose. Points represent mean values obtained from repeated measurements.

The radiation–induced color changes of the PVA/HfO_2_/AgNO_3_ composite thin films were quantified in the CIELAB color space using lightness (L*) and chromatic coordinates (a* and b*) [32]. The primary colorimetric dose metric was the total color difference, ${\Delta E}_{ab}^{*}$. As shown in Figure 3, ${\Delta E}_{ab}^{*}$ increases with absorbed dose over the investigated range. Linear regression over 0–65.2 mGy gives a slope of 0.399 ± 0.022 mGy^−1^ with R^2^ = 0.9845 and a 95% confidence interval for the slope of [0.341, 0.457]. Because the number of dose levels is limited, R^2^ is interpreted descriptively rather than as proof of a universal linear calibration. The L* value decreases from 44.3 at 0 mGy to 20.9 at 57.1 mGy and then increases slightly to 24.0 at 65.2 mGy, indicating an overall dose–associated darkening with some departure from monotonicity at the highest dose (Figure 4). The measured CIELAB parameters were also used to reconstruct the visual color evolution shown in Figure 5. The a* coordinate changes more modestly, while b* decreases over much of the dose range, reflecting a shift in the yellow–blue coordinate (Figure 6 and Figure 7). Taken together with K/S and ${\Delta E}_{ab}^{*}$, the CIELAB parameters demonstrate a consistent dose–dependent optical response of the composite within the investigated range.

### 3.4. Color Strength K/S

**Figure 8:**
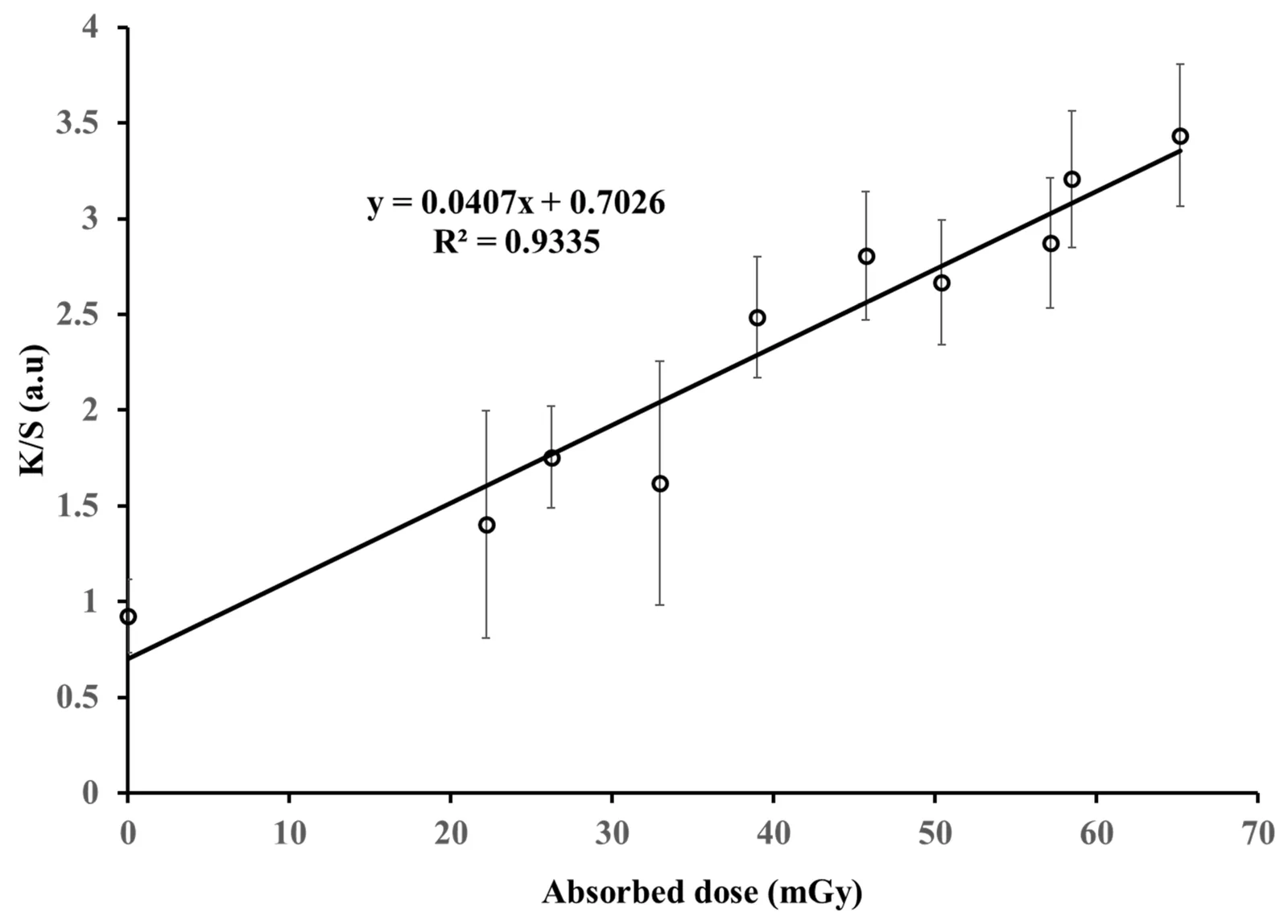
Color strength K/S as a function of absorbed dose for the PVA/HfO_2_/AgNO_3_ composite film. Points represent mean values obtained from repeated measurements. The linear regression is shown over the investigated range.

The Kubelka–Munk color strength (K/S) of the PVA/HfO_2_/AgNO_3_ composite films increases overall with absorbed dose over 0–65.2 mGy, from 0.92 a.u. in the unirradiated film to approximately 3.4 a.u. at 65.2 mGy. Linear regression over the complete dataset gives K/S = 0.0407D + 0.7026 with R^2^ = 0.9335 (Figure 8). This relationship is treated as a descriptive calibration over the measured range. The increasing K/S trend is consistent with the overall decrease in L* and the visible darkening of the reconstructed color swatches because K/S increases as diffuse reflectance decreases. The K/S, L*, and ${\Delta E}_{ab}^{*}$ results therefore provide mutually consistent evidence of dose–dependent optical darkening and support K/S as an additional quantitative response parameter for the composite.

### 3.5. Dose–Dependent CMYK Colorimetric Response

**Figure 9:**
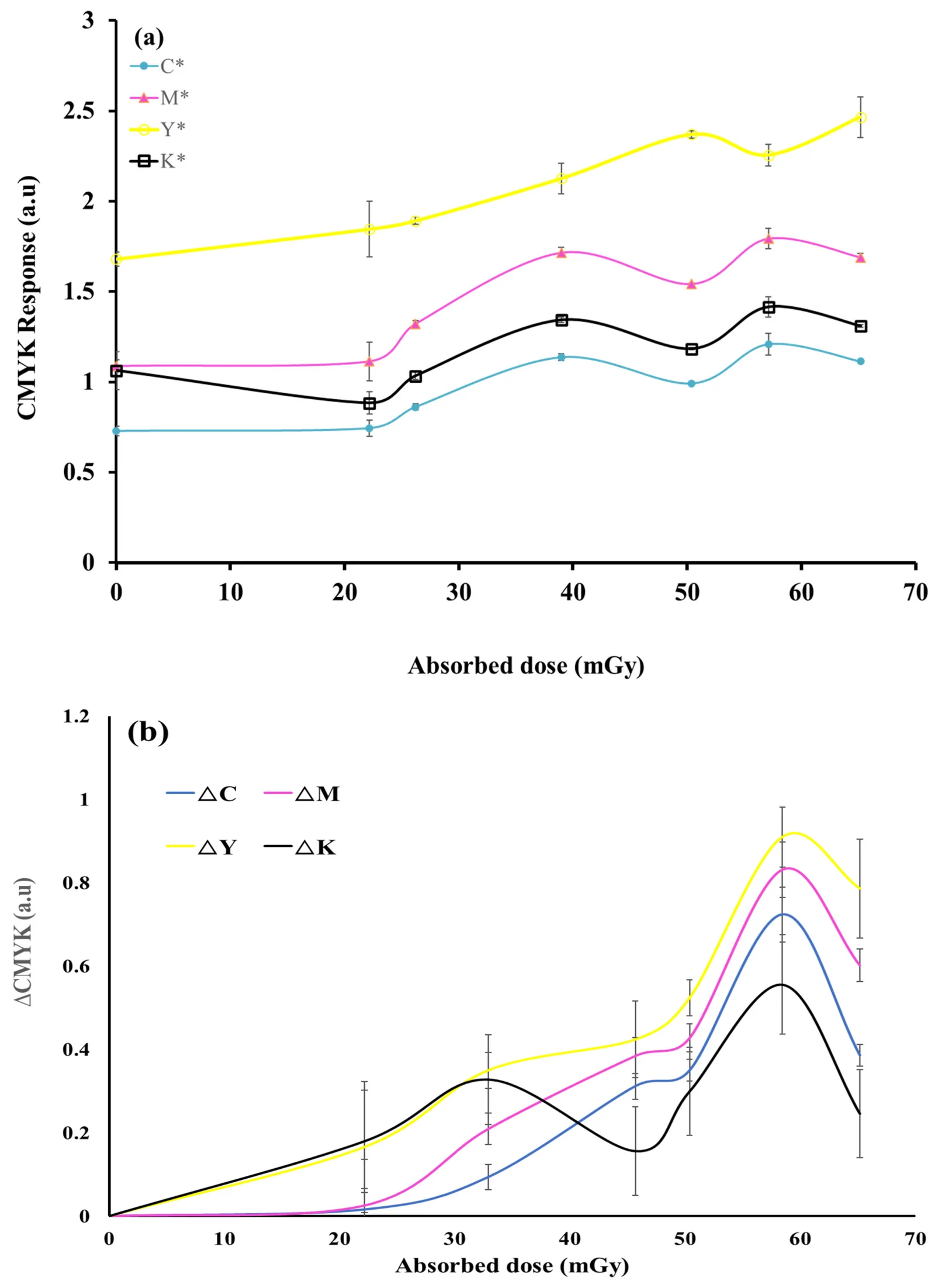
(**a**) Dose–dependent CMYK channel response (C*, M*, Y*, and K*). (**b**) Relative changes in CMYK channels (ΔC, ΔM, ΔY, and ΔK) as a function of absorbed dose for the PVA/HfO_2_/AgNO_3_ composite film under the investigated irradiation geometry.

The relative changes in the CMYK channels (ΔC, ΔM, ΔY, and ΔK) were calculated relative to the unirradiated film, as shown in Figure 9. At lower doses, all four channels increase with dose, while at higher doses, the responses become non–linear. Among the chromatic channels, ΔY shows the largest maximum response (approximately 0.91), followed by ΔM (approximately 0.83) and ΔC (approximately 0.72). The yellow channel is therefore the most responsive of the evaluated CMYK components in the present dataset (Figure 9a). Beyond the region of maximum response, the chromatic channels show a modest decline, consistent with the onset of response saturation or other competing optical changes. The ΔK response is non–monotonic, with a local maximum, a subsequent decrease, and a second increase at a higher dose. Because component–specific control films were not measured, this behavior cannot be assigned uniquely to a particular radiolytic pathway and is therefore treated as an empirical feature of the composite response. Overall, the CMYK analysis supports a dose–dependent color response, with ΔY providing the largest chromatic change over the measured range (Figure 9b).

### 3.6. SEM–EDX Morphological Analysis

**Figure 10:**
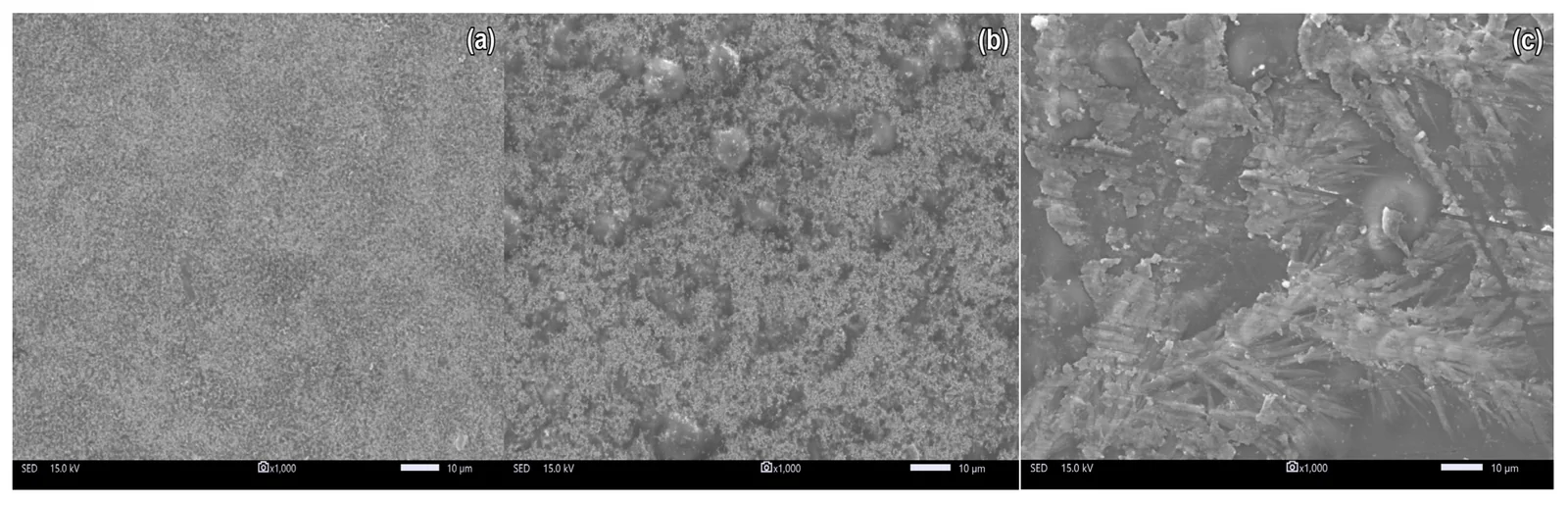
SEM micrographs (×1000 magnification) of the PVA/HfO_2_/AgNO_3_ nanocomposite films: (**a**) unirradiated control (0 mGy), (**b**) film irradiated at 58.4 mGy, and (**c**) film irradiated at 65.2 mGy.

Control and γ–ray–irradiated PVA/HfO_2_/AgNO_3_ nanocomposite films were characterized by SEM–EDX to evaluate the effect of increasing radiation dose on surface morphology and microstructure. The non–irradiated film (0 mGy) displays a uniform, smooth, homogeneous, and compact microstructure without any phase separation, reflecting good compatibility among the constituents of the PVA matrix (Figure 10a). For 58.4 mGy, a clear modification of the surface morphology was observed. The film displays a finer fibrous/spiky texture interspersed with discrete rounded globular aggregates. This indicates the onset of structural reorganization of the polymer matrix and the initial formation of Ag nanoparticles (Figure 10b). The film irradiated at 65.2 mGy exhibits pronounced surface degradation, marked by elongated, tree trunk–like fibrous clusters that radiate outward from flake–like base regions and are separated by visible voids and cavities (Figure 10c). This dendritic, tree–like growth results from the reduction of Ag^+^ ions dissolved within the nanocomposite film, which nucleate and grow into needle– or tree–trunk–like microstructures under irradiation. This dose–dependent morphological degradation appears specific to the PVA/HfO_2_/AgNO_3_ nanocomposite film, suggesting good sensitivity and specificity for use as a dosimeter for low–dose γ–ray detection.

**Figure 11:**
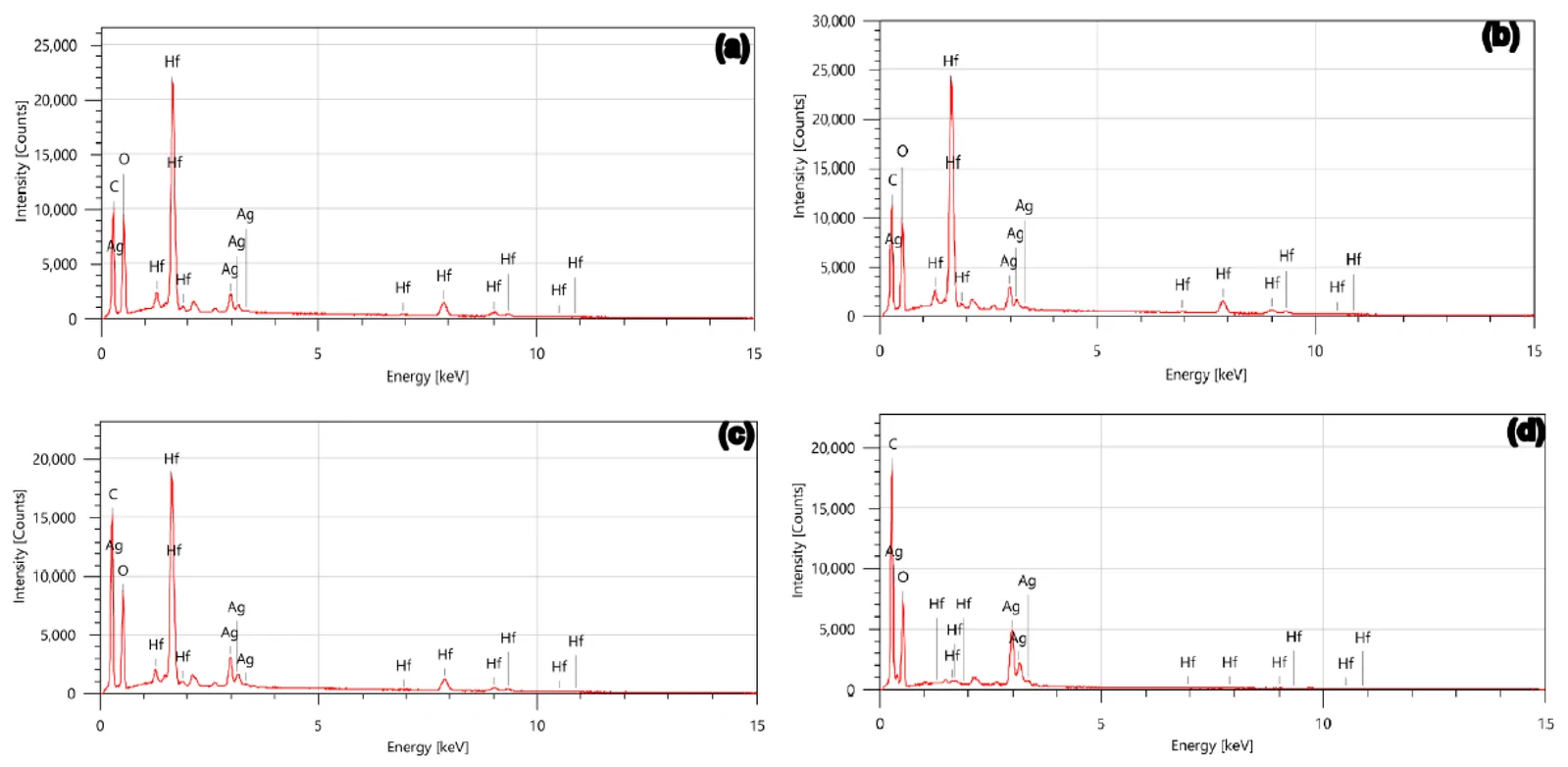
EDX spectra of the PVA/HfO_2_/AgNO_3_ nanocomposite films at (**a**) 0 mGy, (**b**) 22.2 mGy, (**c**) 58.4 mGy, and (**d**) 65.2 mGy γ–ray doses, showing the characteristic C, O, Ag, and Hf peaks used for elemental quantification by EDX analysis.

The EDX elemental analysis showed a different dose–dependent trend in the content of Ag and Hf in the PVA/HfO_2_/AgNO_3_ nanocomposite films, as shown in Figure 11 and Table 4. The mass percentage (m%) of hafnium (Hf) decreased gradually from 45.34% (0 mGy) to 0.84% (65.2 mGy) with increasing γ–ray dose, while the mass percentage (m%) of silver (Ag) increased gradually from 6.01% (0 mGy) to 22.06% (65.2 mGy). This inverse relation suggests that gamma irradiation promotes the surface enrichment of Ag, most likely through radiolytic reduction of Ag^+^ ions from AgNO_3_ to metallic Ag^0^ nanoparticles that gradually shift and subsequently accumulate at the surface of the film. At the same time, the relative decrease in the detected Hf signal with dose may be due to increasing surface coverage by the growing Ag–rich phase, which shields or masks the underlying HfO_2_ nanoparticles from the electron beam, reducing their apparent EDX signal. This dose–dependent change in composition to higher surface Ag content correlates well with the morphological transition to fibrous, tree trunk–like structures observed by SEM, supporting the interpretation of radiation–induced Ag nanoparticle nucleation and growth as the dominant degradation mechanism in the irradiated films.

**Figure 12:**
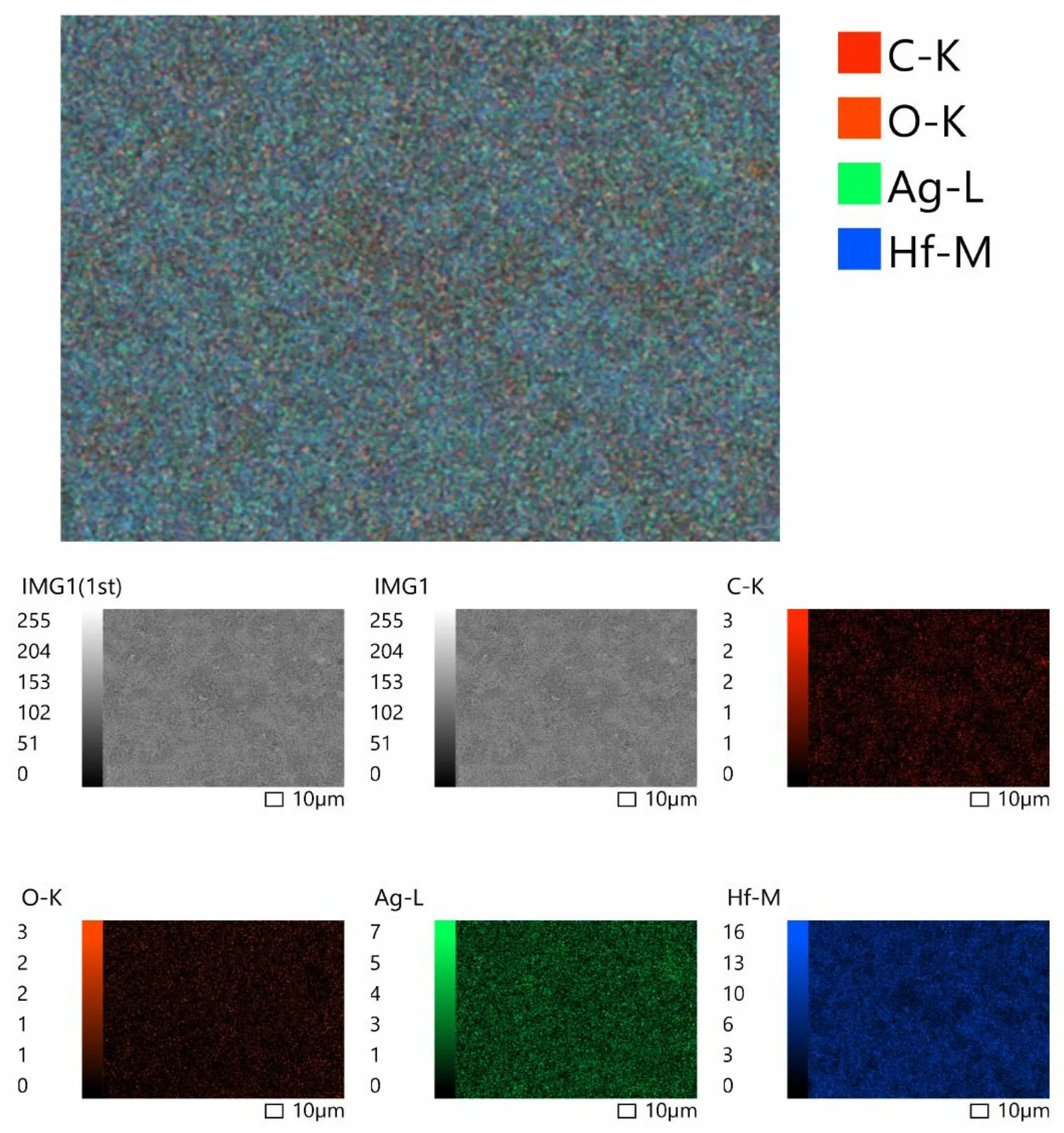
EDX elemental mapping of the PVA/HfO_2_/AgNO_3_ nanocomposite film for a γ–ray dose of 22.2 mGy (×1000 magnification).

The EDX elemental mapping of the nanocomposite film of PVA/HfO_2_/AgNO_3_ was carried out to confirm the uniform dispersion of the added nanoparticles as fillers in the polymer matrix. The composite overlay map (Figure 12) showed the spatial distribution of carbon (C–K, red), oxygen (O–K, orange), silver (Ag–L, green), and hafnium (Hf–M, blue) in the scanned area. The elemental maps exhibit a dense, homogeneous signal distribution across the entire field of view, with no sign of localized clustering, aggregation, or phase segregation. In particular, Ag–L and Hf–M maps show that both AgNO_3_ and HfO_2_ components are homogeneously distributed in the PVA matrix and not accumulated in isolated regions. The uniform distribution shows good compatibility between the polymer matrix and the embedded nanofillers and supports the reproducibility and reliability of the structural and functional properties of the nanocomposite film over the whole surface.

### 3.7. Comparison with Other Dosimetric Materials

The dosimetric behavior of the PVA/HfO_2_/AgNO_3_ film can be placed in context by comparison with established passive and active dosimeters. TLDs and OSLDs offer high sensitivity and broad usable ranges but require dedicated readout systems [7,33,34]. Commercial radiochromic films provide a direct optical record and are a closer functional analogue, although their response and practical dose range depend on film type, beam quality, scanner protocol, and calibration conditions [35]. Electronic detectors such as ionization chambers, semiconductor detectors, Geiger–Müller counters, and scintillation detectors provide real–time or highly sensitive measurements but require powered readout instrumentation [10,36,37]. PVA–based colorimetric systems reported previously also show useful low–dose responses [24,25,27]. In the present study, 22.2 mGy is the lowest tested non–zero dose at which a measurable response was observed; it should not be described as a formal detection limit because a dedicated limit–of–detection analysis was not performed. The principal advantage of the present formulation is its permanent visible response and the possibility of quantitative readout using diffuse reflectance, CIELAB, or image–based color analysis without dedicated electronic detector hardware. Because the irradiation in this study used ^137^Cs gamma rays at 662 keV, direct application to diagnostic X–ray energies requires separate energy–response validation.

### 3.8. Study Limitations

Several limitations should be considered when interpreting the present results. First, an irradiated PVA/HfO_2_ control film without AgNO_3_ was not included. Consequently, although the long–wavelength optical changes are consistent with radiation–induced Ag–containing species based on the present data and published literature, a contribution from irradiation–induced changes in the PVA/HfO_2_ matrix cannot be completely excluded. Likewise, because a HfO_2_–free control formulation was not examined, the individual sensitizing contribution of HfO_2_ cannot be quantified independently from the complete composite response. Second, only four dose levels were available for XRD analysis, so the apparently greater structural change at 65.2 mGy cannot establish a true threshold. These limitations define the scope of the conclusions and identify priorities for future work.

## 4. Conclusions

This study evaluates a PVA/HfO_2_/AgNO_3_ composite thin film as a passive colorimetric dosimeter under low–dose ^137^Cs gamma irradiation. The film exhibits clear dose–dependent optical changes over the investigated 22.2–65.2 mGy range, including decreased reflectance, increased Kubelka–Munk color strength, increased CIELAB total color difference, and measurable changes in the CMYK channels. The ${\Delta E}_{ab}^{*}$ response is approximately linear over the measured range (R^2^ = 0.9845), while ΔY shows the largest relative CMYK response. XRD identifies monoclinic HfO_2_ as the dominant crystalline filler phase and shows dose–associated changes in peak intensity, broadening, and the relative prominence of the polymer–related contribution. These XRD observations are interpreted as structural modification and partial loss of resolved crystalline order rather than definitive evidence for formation of a new crystalline phase. The long–wavelength optical response is consistent with literature describing broadened and red–shifted absorption from Ag–containing metallic species, but the absence of AgNO_3_–free control films prevents exclusive attribution of the response to Ag species. A study of the surface morphology and microstructure of control and γ–ray–irradiated PVA/HfO_2_/AgNO_3_ nanocomposite films was carried out using scanning electron microscopy (SEM–EDX). The transition in morphology to fibrous, tree trunk–like structures identified through SEM is closely linked to the dose–dependent variation in composition favoring higher surface Ag levels, substantiating the idea that the primary degradation mechanism in the irradiated films is the nucleation and growth of Ag nanoparticles induced by radiation. The PVA/HfO_2_/AgNO_3_ nanocomposite films provide a measurable and directly readable colorimetric response in the investigated low–mGy gamma–ray range.

## Figures and Tables

**Table 1:** Composition and functional role of constituents used in the polyvinyl alcohol (PVA)–based composite thin film doped with HfO_2_ and AgNO_3_ for low–dose gamma–ray dosimetry.

Component	wt. (%)	Role in Film	Material Details
HfO_2_	30	High–Z inorganic additive	99.99% purity, 61–80 nm; supplier specification: cubic; US Research Nanomaterials, Inc. (Houston, TX, USA)
AgNO_3_	8	Radiosensitizer colorimetric indicator	Mw = 169.87 g/mol, Sigma–Aldrich (St. Louis, MO, USA)
PVA	62	Polymer matrix and film former	Mw = 85–124 kDa with a hydrolysis degree of 85–90%, Sigma–Aldrich (St. Louis, MO, USA)
Total	100		

**Table 2:** Diffraction angles, d–spacings, Miller indices, and phase assignments for the PVA/m–HfO_2_/AgNO_3_ nanocomposite (0 mGy pattern; Cu Kα, λ = 1.5406 Å). m–HfO_2_ denotes monoclinic HfO_2_ and o–AgNO_3_ denotes orthorhombic AgNO_3_.

2θ°_calc_	2θ°_exp_	d–Spacing (Å)	Miller Indices (hkl)	Reference d (Å)	Assigned Phase
17.56	17.62	5.03	(002)	5.06	o–AgNO_3_
30.17	30.48	2.93	(022)	2.97	o–AgNO_3_
24.25	24.38	3.66	(011)	3.68	m–HfO_2_
28.32	28.44	3.13	(11–1)	3.15	m–HfO_2_
31.68	31.72	2.82	(111)	2.82	m–HfO_2_
34.46	34.46	2.61	(002)	2.61	m–HfO_2_
35.52	35.56	2.52	(200)	2.52	m–HfO_2_
41.05	41.18	2.20	(21–1)	2.18	m–HfO_2_
50.54	50.58	1.80	(220)	1.81	m–HfO_2_
55.74	55.72	1.65	(013)	1.65	m–HfO_2_
60.53	60.54	1.53	(30–2)	1.53	m–HfO_2_

**Table 3:** Summary of the available dose–dependent XRD metrics from the existing diffraction dataset.

Dose (mGy)	Main XRD Behavior	Crystallite–Size Estimate	Representative FWHM/Note
0	m–HfO_2_ reflections dominant; weak 17.62° feature; broad PVA contribution present	~11.6 nm mean HfO_2_ estimate	Baseline d–spacings listed in Table 2
22.2	HfO_2_ peak positions broadly similar to 0 mGy; 17.62° feature no longer resolved	~11.6 nm mean HfO_2_ estimate	No major HfO_2_ change resolved at the lowest dose
58.4	Selected reflections show modest position/intensity changes and broadening	~9.9 nm mean HfO_2_ estimate; ~5.50 nm for the broadened ~51° reflection	FWHM ≈ 1.6° for the ~51° reflection
65.2	Broad PVA–associated ~19.9–20° contribution becomes prominent; several HfO_2_ reflections weaken or are unresolved	3.84 nm is an apparent estimate for the broad ~19.9° feature, not a mean HfO_2_ size	No new crystalline phase is claimed from the available data

**Table 4:** Elemental mass percentage (m%) of C, O, Ag, and Hf in the PVA/HfO_2_/AgNO_3_ nanocomposite films. Values are presented as mean ± standard deviation.

Dose (mGy)	The Element Mass Percentage (m%)			
	C	O	Ag	Hf
0	30.66 ± 0.06	17.99 ± 0.06	6.01 ± 0.05	45.34 ± 0.14
22.2	30.66 ± 0.05	17.44 ± 0.06	7.54 ± 0.05	44.37 ± 0.13
58.4	38.67 ± 0.06	17.62 ± 0.07	8.23 ± 0.05	35.47 ± 0.12
65.2	43.96 ± 0.06	33.13 ± 0.13	22.06 ± 0.10	0.84 ± 0.05

## Data Availability

Data available in a publicly accessible repository: The data presented in this study are openly available in Zenodo at [https://doi.org/10.5281/zenodo.21549767].
